# Effects of Blood Contamination and Decontamination Protocol on Reverse Torque Value of Abutment Screws in Dental Implants: An In Vitro Study

**DOI:** 10.3390/biomimetics8020157

**Published:** 2023-04-14

**Authors:** Hafiz A. Adawi, Harisha Dewan, Arwa Khawaji, Hadeel Akkam, Areej Hakami, Bashair Wasli, Maram Hakami, Maimonah Alali, Hitesh Chohan

**Affiliations:** 1 Department of Prosthetic Dental Sciences, College of Dentistry, Jazan University, Jazan 45142, Saudi Arabia; 2 General Dentist, College of Dentistry, Jazan University, Jazan 45142, Saudi Arabia; 3 Dental Intern, College of Dentistry, Jazan University, Jazan 45142, Saudi Arabia; 4 Department of Restorative Dental Sciences, College of Dentistry, Jazan University, Jazan 45142, Saudi Arabia

**Keywords:** dental implant, abutment screw, blood decontamination solution, reverse torque value

## Abstract

*Background and Objective*: Loosening of abutment screws in dental implants is a mechanical complication that affects prosthetic treatments and hence, patient satisfaction. Blood contamination of abutment screws may play a role in this phenomenon. However, only limited research attention has been given to this issue. In the present study, we determined the effect of blood contamination and decontamination protocol on the reverse torque value (RTV) of abutment screws. *Materials and Methods*: A questionnaire-based survey was sent to 210 implantologists requesting feedback on their attitude to the blood contamination issue and the decontamination protocols used. The survey responses were used in a selection of the decontamination solutions that were used in the subsequent in vitro study on the effects of blood decontamination protocol on the RTV of abutment screws. Thus, three study groups were used (*n* = 20 abutment screws in each group): Group 1 (control group; blood-contaminated screws); Group 2 (screws decontaminated with 5.25% sodium hypochlorite (NaOCl) solution); and Group 3 (screws decontaminated with normal saline solution (0.9%)). Then, each of the connections were subjected to thermocycling, and RTVs of the screw were measured using a digital torque meter. Intragroup and intergroup RTVs were analyzed for significance using analysis of variance (ANOVA) and Tukey’s honestly significant difference (HSD) tests. *Results*: 48% of the implantologists responded to the survey; 80% of them were concerned with blood contamination in the implant connection, especially before abutment loading and 85% of them used either chlorhexidine solution or normal saline solution as the decontamination agent. The mean RTV for Group 2 screws (30.27 ± 2.8 N.cm) was significantly greater than that for Group 3 screws (26.02 ± 1.99 N.cm) which, in turn, was significantly greater than that for Group 1 screws (23.64 ± 1.84 N.cm). *Conclusion*: Decontamination of blood-covered connections using 5.25% NaOCl solution or normal saline solution restores the RTV of abutment screws. This finding may have clinical relevance in that the decontaminated screws may contribute to the low incidence of screw loosening and, ultimately, improved patient satisfaction.

## 1. Introduction

In recent years, the rate of clinical success of dental implants has increased markedly, which is a reflection of the increase in the number of implantologists with the requisite experience in performing the procedure [1,2,3,4]. Both biological and mechanical issues might contribute to an implant failing. While mechanical causes primarily involve prosthetic components, such as overloading of the prosthesis –implant–pillar complex, implant fracture, abutment fracture, loosening of the screws, fracture of the superstructure (metal/ceramic), and biological causes primarily concern periimplantitis, which affects the soft and hard tissues around the implants. Despite this, many studies have reported biological and mechanical failures of dental implants [5,6,7,8]. Common causes or contributing factors include mechanical retention issues, loosening of prosthetic or abutment screws, problems with the opposing dentition, (such as supra-occlusal contacts and damage to opposing overdentures), and fractures of assembly components [7,8,9]. The abutment screw joins a prosthetic abutment to the implant body. A clamping force is generated during the elastic recovery of the elongated screw, which helps to hold the abutment and implant together. The preloading required is influenced by many variables, such as design and number of threads in the implant and contamination of the screw head by blood.

A dental implant complex’s joint design aims to create a tight connection between all of the assembly pieces and to create enough resistance to outside influences. The torque applied to tighten the screw’s head has an impact on the resistance by compression between the appropriate threads of the abutment screw and implant, as well as at the interfaces where the screw head and abutment meet. In order to withstand dynamic loading and prevent screw loosening, the ensuing tensile force inside the abutment screw, known as the “preload,” is equivalent to a stress in the screw that should be between 60% and 75% of the material’s yield strength. Preload, the impact of settling, and screw shape can all have an impact on the stability of abutment screws. The movement that results from applying tangential force to the screw is known as torque, and it is often denoted in newton centimeters (N.cm). Because the implant–abutment junction’s components are absorbing the majority of the load when preload is applied to a screw, the connected elements are retained in compression and the screw experiences only minor impacts. Typically, a torque wrench is used to provide torque to the screw in order to insert the first preload. The “loss of preload” is one of the key contributors to screw loosening [10,11,12,13,14,15].

Screw loosening can result in a number of clinical issues, including prosthesis displacement and loss of function [16,17,18,19,20,21,22,23]. Irritation and pain as a result of screw loosening are frequently experienced at the interface of the implant and the abutment in deeper regions. Additionally, screw loosening expands the micro-gap of the implant assembly, and a larger gap may compromise the integrity of the implant [16,17]. The success of the implant is influenced by the ergonomics of the implant screw and also by the clinical procedure. Screw loosening is still the most common issue, especially in single crowns. According to the claim made by authors of a previous study, screws with higher preloads require stronger forces to be removed. Yet, other researchers asserted that even after applying the necessary tightening torque values, micro-gaps remain between these surfaces and they cause unintended motions, leading to screw-related mechanical problems. These openings also offer places where microorganisms can survive. Their byproducts migrate into the tissues around the implant [16]. As a result of these microbiological activities, a slippery environment that affects screw loosening may develop. It is ultimately impossible to stop periimplantitis with the peak of inflammatory cell content, particularly around the implant–abutment contact caused by the misfit of these surfaces. The amount of torque applied, the macro- and micro-geometry of the implant, the component position, the device used for applying torque, presence of cantilevers, type of occlusal table, cuspal anatomy, bone quality, direction of forces, and presence or absence of parafunctional activities are among the factors that affect the preload and which eventually lead or contribute to screw loosening. Screw loosening may be a significant factor in the rise in referrals for implant care, which is time-consuming and uncomfortable for the patient. During clinical and experimental operations, blood, saliva, fluoride, and chlorhexidine could contaminate the implant screw hole. During placement of the implant, restoration of the prosthesis, the components of the implant, including the cover screw, are inserted and removed several times from the implant body. Implant surgeries, making impressions, and abutment trials may lead to the contamination of the screw hole with blood. Many investigations examining the behavior of abutment screws following loading have been conducted during the past few years. However, in spite of the high likelihood for the contamination of the abutment screw hole during surgical and try-in visits, there are only a few studies in the literature on the effect of blood contamination on the implant–abutment reverse torque value (RTV) and the best protocol for the decontamination of the screw hole during the implant procedure. 

The purpose of the present study was to fill this knowledge gap. Specifically, we determined the effect of two widely used decontamination solutions, sodium hypochlorite (5.25%) and normal saline (0.9%), on the (RTV) of abutment screws. Additionally, the morphologies of the surfaces of the screws at the end of each test were examined using a scanning electron microscope. Our study’s null hypothesis was that the RTV of the contamination groups and the control group did not differ in a manner that was statistically different from one another. The data collected can provide clinicians new knowledge with regard to the impact of potential fluid contaminations at the implant–abutment contact.

## 2. Materials and Methods

### 2.1. Questionnaire-Based Survey

The Ethics Committee approved the study (Reference no. CODJU-21071; 11 November 2021). 

A questionnaire-based survey on implant connection contamination and decontamination protocols was created and sent (via e-mail and social media platforms) to 210 dental implantologists to obtain information about their awareness of implant connection contamination and their use of decontamination protocols. The subject, types of participants, the purpose of the survey, and deadlines were clearly mentioned in the survey link. The survey questions were as follows:

1. How many years of clinical experience do you have?
2. At the time of dental implant placement, are you concerned if the implant connection gets contaminated with blood?
3. At the time of dental implant loading, are you concerned if the implant connection gets contaminated with blood?
4. If you are concerned with implant connection contamination, please specify the decontamination protocol(s) you use.

After the survey results were received, an in vitro study was conducted in the Phantom Laboratories at the College of Dentistry of Jazan University, using Grade 5 titanium implants (TRI Dental Implants Int. AG, Bösch, Hünenberg, Switzerland). The specifications of the implants were as follows: 4.7 mm diameter; 3.5 mm prosthetic platform with 2.5 mm internal hexagon; 16 mm length; integrated triple-lead-thread; sandblasted surface, medium roughness; and 0.5 mm machined shoulder. 

### 2.2. Sample Size Calculation

Fourteen implants were used in a pilot study, and the sample size was calculated [24]. Two study groups were used for the pilot study: Group 1 had blood-contaminated screws and Group 2 had decontaminated screws. Each group consisted of 7 implants. The sample size formula was as follows:

n = [2S^2^(Zα + Z_β_)^2^]/d^2^

             = [2 × 9.7 (1.96 + 1.04)2]/(2.98)2 = 174.6/8.88 = 19.66

where Zα is the Z-value for the α level (1.96 at 5% α error or 95% confidence), Zβ is the Z- value for the β level (1.0370 at 15% β error or 85% power), d is the margin of error (2.98%), and S is the pooled standard deviation, (S1 + S2)/2, where S1 was 1.3 and S2 was 1.8. Thus, in the vitro study, the number of implants to be used was 20.

### 2.3. Specimen Preparation

**Figure 1:**
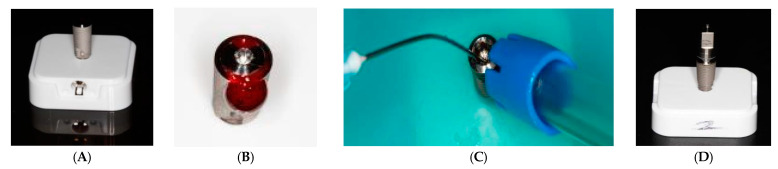
(**A**) The implants attached to the plastic base. (**B**) The abutment connection contaminated with blood donated by one of the authors. (**C**) The implant connection closed with cover screws and disinfected using CaviCide spray. (**D**) Each implant was placed in the plastic box for seven days in a humid incubator.

A total of 60 implants were used. Each of the implants was attached to the plastic base provided with the implants from the manufacturer (Figure 1A). The abutment connections were contaminated with blood (Figure 1B) donated by one of the authors after he/she signed an informed consent form. Blood was collected intravenously (approximately 2 mL) and the implants were contaminated before the coagulation time with a pipette until the blood filled the inner surface of the implant fixture to replicate the oral environment. After contamination with blood, the implant connections were closed with cover screws and then disinfected using CaviCide spray (Metrex Research, Romulus, MI, USA) (Figure 1C) as a standard protocol for all the implants. Each implant was then placed in a plastic box for seven days in a humid incubator (Figure 1D). The implants were divided into 3 groups (20 implants per group). The cover screws were then removed and the implant abutment connection was decontaminated using different protocols (except for the Control Group). The 3 study groups were as follows:

Group 1: control group (blood-contaminated implants);

Group 2: implants decontaminated using 5.25% NaOCl (Dentaflux, Algete, Madrid, Spain) for 30 s, followed by a 30 s waiting period, and then irrigated with 0.12% chlorhexidine gluconate for 30 s;

Group 3: implants decontaminated using normal saline (0.9%) (Pharmaceutical Solutions Industry, Jeddah, KSA) for 30 s, followed by a 30 s waiting period, and then irrigated with 0.12% chlorhexidine gluconate for 30 s.

### 2.4. Torque Measurement

**Figure 2:**
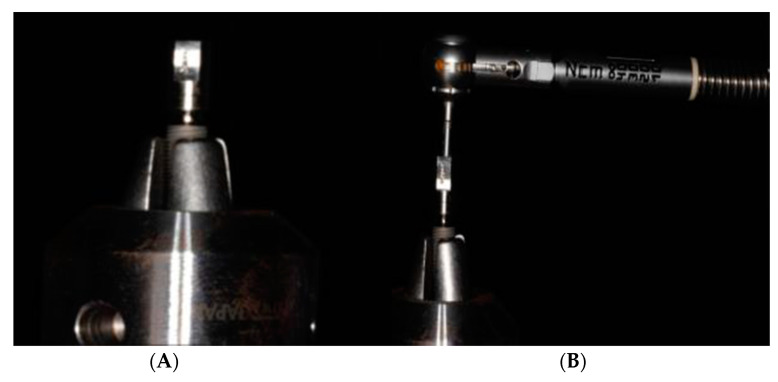
(**A**) Implants mounted on the holder of a digital torque meter. (**B**) The abutment screw was preloaded to 30 N.cm using a manual torque ratchet.

The implant connections were dried with paper points. A straight abutment was attached to each implant connection and then the implants were mounted on the holder of a digital torque meter (TQ-8800, Lutron Electronic Enterprise Co., Taipei, Taiwan) (Figure 2A). The recording of pure torque was achieved by applying various transverse forces equivalent to those used by the wrench, and then the torque meter was calibrated so that the torque meter became insensitive to these transverse attempts. An initial torque value (ITV) of 30 N.cm was applied with a handheld torque wrench (Figure 2B), as recommended by the implant manufacturer. A force was applied until the digital torque meter screen reached the value of 30 N.cm. The zero key was then pressed to allow the torque meter to take fresh measurements. The ITV was thus kept constant for all the test implants (30 N.cm).

The implant and abutments were subjected to thermocycling in a thermocycling unit (Model 1100, SD Mechatronik, Bayern, Germany) from 5 °C to 55 °C for up to 5000 thermal cycles (dwell time of 30 s), which simulated a period of 6 months of clinical service [25]. The implants were then remounted on the holder of the digital torque meter and the same operator recorded the RTVs for each mounted implant-screw pair.

#### Scanning Electron Microscope (SEM) Analysis

A scanning electron microscope (LEO 440 Computer Controlled Digital Scanning Electron Microscope, Cambridge, UK) at 20 kV (339× magnification) was used and one abutment from each of the three groups was analyzed. Though the first investigator was aware of the group allocation (not blinded), groups were coded and the analysis was carried out by the second investigator (blinded).

### 2.5. Statistical Analysis

The survey responses and the results of the in vitro study were evaluated using Statistical Product and Service Solutions version 21 software package (SPSS Inc., Chicago, IL, USA). The intra- and intergroup values were analyzed for significance using the Kruskal–Wallis ANOVA test followed by Mann–Whitney U test for calculating the level of significance, with *p* < 0.05 denoting significance.

## 3. Results

**Figure 3:**
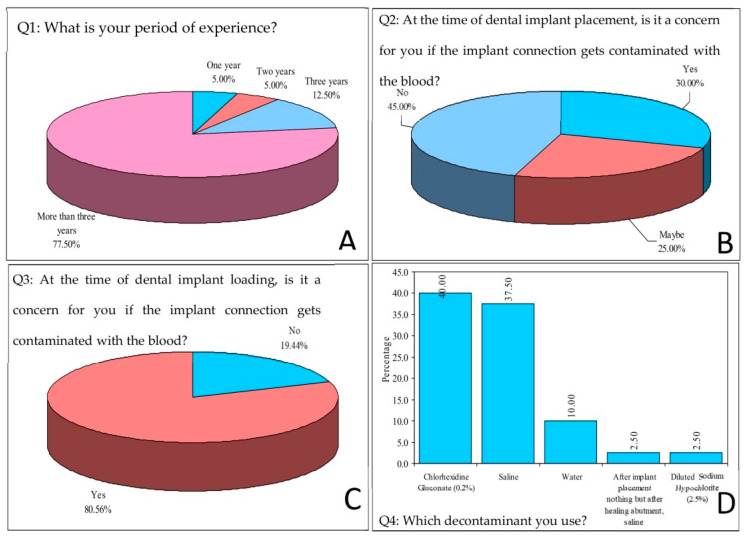
Analysis of survey responses. Responses for (**A**) Period of experience of the participant, (**B**) Concern regarding the contamination of the implant connection with the blood at the time of dental implant placement, (**C**) Concern regarding the contamination of the implant connection with the blood at the time of dental implant loading, and (**D**) Specification of the material for decontamination.

The survey results show that most dental implantologists were concerned about the contamination of the implant connection with blood, especially prior to abutment loading (Figure 3B,C). Additionally, a large majority of the dental implantologists who responded (80%) used chlorhexidine or normal saline as the decontamination agent (Figure 3D).

In ascending order of magnitude, the RTVs (Table 1) were Group 2 > Group 3 > Group 1. The mean RTVs of Group 1 (23.64 N.cm) and Group 3 (26.02 N.cm) were lower than the ITV (30 N.cm).

It was noted that the RTV scores in Group 1 and Group 3 did not follow a normal distribution (Table 2). Therefore, the non-parametric tests, i.e., Kruskal–Wallis ANOVA test followed by Mann–Whitney U test, were carried out (Table 3).

A significant difference was observed between three groups (Group 1-3) with mean RTV (*p* < 0.05) at 5% level of significance. This means that the RTV scores are different in three groups (Group 1-). Further, to know the pair wise comparisons, the Mann–Whitney U test was applied and the results are presented in the following Table 4.

**Figure 4:**
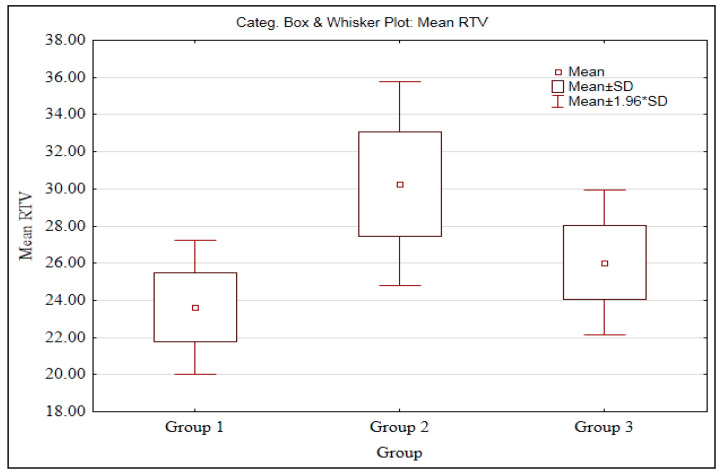
Box plot of the RTVs.

A significant difference was observed between Groups 1 and 2, Groups 1 3, and Groups 2 3 (*p < 0.05*) which indicates that the mean RTV scores are significantly higher in Group 2 as compared to Group 1, in Group 3 as compared to Group 1, and in Group 2 as compared to Group 3. The mean scores are also presented in the box plot (Figure 4).

**Figure 5:**
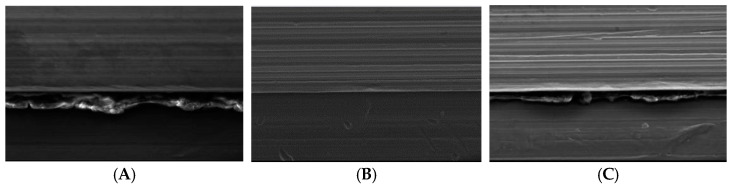
Scanning electron micrographs of an abutment screw specimen from Group 1 (**A**), Group 2 (**B**), and Group 3 (**C**).

A prominent layer (bio- film) could be seen on Group 1 specimens (Figure 5A). Group 3 specimens also showed a biofilm layer, although it was not very prominent (Figure 5C). No such layer was evident on Group 2 specimens (Figure 5B).

## 4. Discussion

Despite the fact that blood can enter the abutment screw hole during surgery and the try-in stages for bone-level implants, very few previous studies in the literature focused on the contamination of the abutment screw hole with blood. The present study began with a questionnaire that was sent to 210 practicing dental implantologists, which was met with a good response rate of 48%. The purpose of the questionnaire was to know the decontaminant protocol that the clinicians use for decontamination of the abutment screw hole, because there is a very limited literature to support protocol selection. The survey results showed that chlorhexidine and normal saline solutions were the decontaminants used most often. In the in vitro study, the effect of blood contamination and the application of the two aforementioned decontaminants on RTV were investigated.

When a torque is given to the screw head, the preload acts as strain on the screw to keep its parts from separating. A total of 90% of the initial torque is needed to overcome friction caused by surface defects, while only 10% is transformed into preload. Preload is a force inside the screw that is produced by the applied torque. The screw’s preload, which is directly proportional to the insertion torque, creates a clamping force between the screw and the Ti-base to prevent external forces from separating the screw and Ti-base. To keep the screw from giving way or breaking, the preload force must be within the elastic limit and not too high. According to earlier research, the optimal preload for abutment screws is between 60 and 75 percent of their elastic limit [26,27,28]. The suggested torque value varies depending on the manufacturer; for the current system, it is 30 N.cm.

Implant connection geometries with various mechanical, biological, and esthetic properties have been created recently. There are two fundamental geometries: internal connections and external connections. Under conditions of high occlusal loads, the external hexagon may permit microscopic abutment movement, leading to joint instability that could lead to abutment screw loosening or even fatigue fractures [29,30,31]. According to the literature, this form of connection loosens up between 6% and 48% of the time, creating a mechanical challenge for maintaining preload (torque for removing the pillar must be 10% less than that for installation) [13]. The present implant study used internal hexagon connection. The internal connection made with a Morse cone results in a more precise binding between the implant and the abutment, which lessens interface movement and the loosening of the screw (the torque required to remove the abutment must be 17% more than that required to install it) [13]. An internal cone of 8° or 11° on the Morse cone helps prevent screw loosening [9,16].

There are currently two main methods for calculating preload. Strain gauges are used to measure the tension when the abutment screw is secured to the implant fixture; however, this takes a lot of time and effort. The alternative method is RTV measurement. The amount of effort needed to rotate an abutment screw in the opposite direction is known as the reverse torque value. The amount of tension still present on the screw that may generate friction and so prevent the screw from loosening is indicated by the reverse torque value of the screw. The bulk of studies have demonstrated a direct relationship between tightening torque value and preload, hence the current study makes use of RTV measurement to predict preload changes. A digital torque meter that had previously been calibrated and had an accuracy of ±0.3%, was used to measure the torque values.

There are very few investigations on the blood contamination of the abutment screw hole in the literature, despite the relatively significant risk of blood contamination of the abutment screw hole in clinical practice, especially for bone-level implants during surgery and try-in stages. Blood contamination may cause a thin layer to form on the surface of titanium implants due to the high protein content and the presence of macromolecules such as fibrinogen and platelets. According to previous studies, as soon as blood comes in contact with a metal surface, platelets begin to interact with it [32].

The mean RTV score for Group 1 (with only blood contamination and no decontamination) and Group 3 (decontaminated using normal saline) were less than the initial ITV. This was partially in accordance with the findings by Gumus et al., who concluded that the blood contamination significantly reduces RTV [20]. Blood contamination thus reduces the screw preload torque value, which may initiate or accelerate the loosening of the abutment screw. The settling effect and friction may be responsible for the drop in torque value. It was estimated that the settling effect will result in a loss of 2–10% of the initial preload. The micro-roughness loss of the screw thread and internal surface during loading is what leads to screw settling. If functional loading is equivalent to or greater than the screw’s preload, it may result in screw loosening. Clinically applied screw torque can act as a clamping force on the screw and abutment [26,27]. The gripping force may decrease and the screw may release if the torque drops below the manufacturer’s suggested level. The present study showed that the drop in ITV in the control group is more than 10–15% and hence, is a cause of concern.

Gumus et al. also reported that there was no significant effect on RTV when chlorhexidine was used before implant insertion. Hence, for our study, a commercially available form of chlorhexidine gluconate (0.12% concentration) was used as an irrigant after decontamination with either NaOCl solution (Group 2) or normal saline solution (Group 3). Because this is the most commonly used irrigant in dental clinics, no separate procurement of the solution was needed. The use of these solutions therefore increases the ease of application and level of convenience, as suggested by the results of the survey questionnaire distributed to the implantologists.

The present results suggest that the most effective decontamination solution for blood in the implant connection was irrigation with 5.25% sodium hypochlorite for 30 s followed by flushing irrigation with 0.12% chlorhexidine gluconate for 30 s. Our findings ruled against the null hypothesis.

Previous researchers rarely evaluated the decontamination of implants soiled with blood using combinations of 5.25% NaOCl with chlorhexidine gluconate or of saline with chlorhexidine gluconate. Hence, the literature results are very small in amount [5,20,22,32], to which the present ones may be compared. We found that relative to ITV, RTV for the control and normal saline group were each lower, but that for the NaOCl 5.25% group was about the same. Gumus et al. compared implants contaminated with blood, saliva, and chlorhexidine with uncontaminated specimens and concluded that blood contamination resulted in the lowest RTV [20]. Similarly, Micarelli et al. found higher RTV values compared with their control groups. However, they used a chlorhexidine gluconate gel [22]. Tzenakis et al. concluded that the effects of saliva were similar to those of contamination with blood with increasing preload values [5]. Proteins and macromolecules, such as platelets and fibrinogen, can result in blood build-up on the surface of titanium implants, which can cause them to break down and leak. Metal surfaces and blood begin to interact with each other as soon as they come in contact with platelets. A biofilm build-up on the abutment screw surface may impair RTV, which is a finding similar to that of Christersson et al. [32]. The results of this study imply that the buildup of a biofilm on the surface of the abutment screw, as evident in the SEM analysis, may be detrimental to RTV. The decrease in RTV seen after contamination in Group 1 and Group 3 may be significantly influenced by blood viscosity.

Our findings may guide clinicians in the selection of a blood decontamination protocol that restores initial torque on abutment screws. In turn, this may reduce the incidence of abutment-screw failures, which may lead to better clinical outcomes for dental implants and hence, increased patient satisfaction.

Apart from the usual limitations of an in vitro study, other study limitations are: (1) only two decontamination protocols were used and (2) the sample size for each study group (*n* = 20) was small—this being a consequence of the small budget available for the study. Additionally, it is recognized that other body fluids apart from blood, such as saliva, may contaminate the implants. Furthermore, the clinical outcome of a dental implant is influenced by a large number of factors, such as patient dentition in general, the technique used by the implantologist, implant type, and dental loading pattern (especially repeated loadings). In our study, contaminations could develop at the implant–abutment connection following abutment torqueing during prosthesis function; consequently, it is advised that future research look into the impact of contaminating implant–abutment specimens with various substances while subjected to cyclic loading.

## 5. Conclusions

1. The RTV of Group 1 implants (23.64 ± 1.84 N.cm) was significantly lower than the ITV (30 N.cm). Thus, blood contamination significantly lowers RTV.
2. RTVs for the study groups, from the highest to lowest, were as follows: Group 2 > Group 3 > Group 1. Irrigation with 5.25% sodium hypochlorite followed by flushing with 0.12% chlorhexidine gluconate at the implant–abutment connection removes a substantial amount of blood contamination and maintains the initial torque value.

The present results support the recommendation that dental implantologists must clean the abutment screw hole after it has been contaminated with blood during various implant procedures.

## Figures and Tables

**Table 1:** Summary of RTV values (in N.cm).

Groups	Mean	Standard Deviation	Standard Error	95% Confidence Interval of Mean	
				Lower Bound	Upper Bound
Group 1	23.64	1.84	0.41	22.78	24.49
Group 2	30.27	2.80	0.63	28.95	31.58
Group 3	26.02	1.99	0.44	25.09	26.95

**Table 2:** Normality of RTV scores in three groups (Group 1, 2, 3) by Kolmogorov–Smirnov test.

Groups	z-Value	*p*-Value
Group 1	0.2570	0.0010 *
Group 2	0.1480	0.2000
Group 3	0.2660	0.0010 *

* *p* < 0.05, statistically significant.

**Table 3:** Comparison of three groups (1, 2, 3) with RTV scores by Kruskal–Wallis ANOVA.

Groups	Mean	SD	Mean Rank
Group 1	23.64	1.84	13.35
Group 2	30.27	2.80	47.85
Group 3	26.02	1.99	30.30
H-value	39.1870		
*p*-value	0.0001 *		

* *p* < 0.05, statistically significant.

**Table 4:** Pair-wise comparison of mean RTV scores of the three groups (Group 1-3) by Mann–Whitney U test.

Groups	Z-Value	*p*-Value
Group 1 vs. Group 2	−5.0980	0.0001 *
Group 1 vs. Group 3	−4.2110	0.0040 *
Group 2 vs. Group 3	−4.3220	0.0001 *

* *p* < 0.05, statistically significant.

## Data Availability

The data that support the findings of this study are available from the corresponding author upon reasonable request.
